# Genetic variation in bitter taste receptor genes influences the foraging behavior of plateau zokor (*Eospalax baileyi*)

**DOI:** 10.1002/ece3.2041

**Published:** 2016-03-06

**Authors:** Fang Zhao, Tongzuo Zhang, Jiuxiang Xie, Shoudong Zhang, Eviatar Nevo, Jianping Su, Gonghua Lin

**Affiliations:** ^1^ Key Laboratory of Adaptation and Evolution of Plateau BiotaNorthwest Institute of Plateau BiologyChinese Academy of SciencesXining810001China; ^2^ University of Chinese Academy of SciencesBeijing100049China; ^3^ Department of Grass ScienceQinghai UniversityXiningQinghai810016China; ^4^ Institute of EvolutionUniversity of HaifaHaifa3498838Israel

**Keywords:** Bitter taste, food selection, single‐nucleotide polymorphism, subterranean rodent

## Abstract

The ability to detect bitter tastes is important for animals; it can help them to avoid ingesting harmful substances. Bitter taste perception is mainly mediated by bitter taste receptor proteins, which are encoded by members of the *Tas2r* gene family and vary with the dietary preference of a specific species. Although individuals with different genotypes differ in bitterness recognition capability, little is known about the relationship between genetic variation and food selection tendencies at the intraspecific level. In this study, we examined the relationship between genotypes and diet in plateau zokor (*Eospalax baileyi*), a subterranean rodent endemic to the Qinghai‐Tibet Plateau that caches food for the winter. We assayed the composition and taste profile of each plant contained in temporary caches and vicinity quadrats, which were representative of selected and available food, respectively. Bitter plant selection indices (*E*_bitter_) were estimated. We also sequenced 26 candidate *Tas2r* genes from zokors and determined their relationships with the *E*_bitter_ of their caches. We identified four key results: (1) zokors varied considerably in both bitter food preference and *Tas2r* sequences; (2) five genes (*zTas2r115*,*zTas2r119*,*zTas2r126*,*zTas2r134*, and *zTas2r136*) exhibited allelic variation that was significantly associated with *E*_bitter_; (3) synonymous SNPs, nonsynonymous SNPs, and pseudogenization are involved in the genotype–phenotype relationship; (4) the minor genotypes of *zTas2r115*,*zTas2r134*, and *zTas2r136* and the major genotypes of *zTas2r119* and *zTas2r126* cached more bitter plants. Our results link *Tas2r* variation with food selection behavior at the population level for the first time.

## Introduction

Vertebrates can typically perceive five distinct tastes: sweet, umami, bitter, sour, and salty. Bitter tastes are particularly important for animals because they are often associated with potentially harmful substances (Mueller et al. 2005). Bitter tastes are chiefly mediated by the bitter taste receptor proteins encoded by members of the *Tas2r* gene family (Shi et al. 2003). A striking pattern has emerged among species; the diet of a species shapes the repertoire of *Tas2r* genes that are contained in its genome (Jiang et al. 2012; Li and Zhang 2013; Zhao et al. 2015). In humans, different genotypes confer different bitterness recognition sensitivities (Sandel and Breslin 2006). However, difficulties in characterizing the full dietary range of wild animals have made it difficult to identify relationships between genetic variation and food selection tendencies at the intraspecific level.

**Figure 1:**
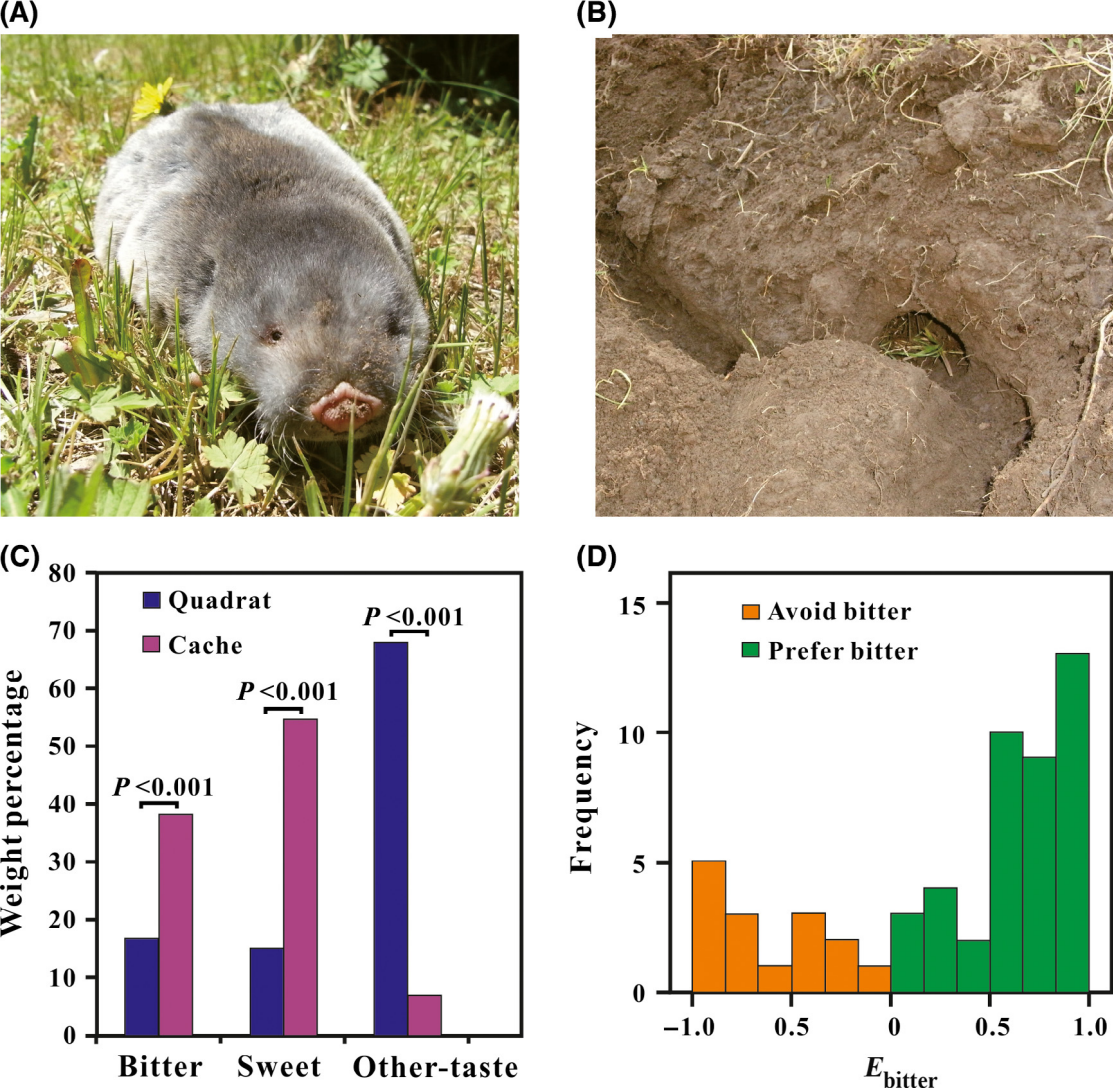
(A) frontal view of a plateau zokor; (B) a cache in the zokor burrow system; (C) weight percentages (average) of plants with different tastes to humans in quadrats and caches (*P* values showed two‐related samples Wilcoxon signed ranks test between quadrats and caches); (D) *E*_bitter_ (selection index of bitter plants) distribution of the surveyed zokors (*N *=* *56).

Subterranean rodents comprise a widely distributed collection of taxa that live primarily underground and are highly adapted to their environments (Nevo 1999). The subterranean niche protects subterranean rodents from both predators and the environmental fluctuations that predominate above ground. However, these animals typically have extremely diminished vision (Begall et al. 2007); hence, directly tasting (and probably also smelling) plants in the dark burrows may be fundamental to effective foraging. Plateau zokors (Rodentia, Spalacidae, *Eospalax baileyi*) (Fig. 1A) are a typical subterranean rodent in the Qinghai–Tibet Plateau (QTP). They spend most of their life underground and collect nearly all of their food resources (either underground organs or whole plants) through digging activities. Soil in the QTP is frozen solid throughout the coldest half of the year, and during this time, plateau zokors are unable to forage. Each autumn, these animals store food in their caches for the approaching and long cold season (Fig. 1B). The caching behavior of plateau zokors enables researchers to accurately characterize diet selection in the field by surveying their cache composition. Our previous studies demonstrated that plateau zokors do not forage randomly when collecting food for winter caches (Xie et al. 2014a,b). Rather, these animals generally prefer sweet and bitter plants while avoiding plant materials characterized by other tastes (including tasteless, pungent, sour, and other tastes that are difficult to describe but do not taste bitter at all; Xie 2014), indicating that taste plays an important role in food selection behaviors.

For a given target plant, individual animals exhibited distinct preferences (Kim et al. 2005; Xie 2014). Although *Tas2r* genotype information was previously unavailable for plateau zokor, high levels of *Tas2r* diversity within human (Kim et al. 2005) suggest that zokors may also differ in *Tas2r* genotypes and may thus exhibit variable bitter plant preferences. In this study, we assayed the compositions and taste profiles of the plants contained in zokor caches and in quadrats in the vicinity of caches, which represented the total available food sources. This information was used to calculate bitter plant selection indices for individual zokors. We also sequenced all *Tas2r* genes of individual animals to determine their genotypes. These data allowed us to characterize the relationship between *Tas2r* genotypes and bitter preference phenotypes at the intraspecific level.

## Materials and Methods

### Food selection measurements

The study was conducted in Xinzhuang, Datong County, Qinghai Province, China (37°30′ N, 101°13′ E, elevation ~3000 m above sea level). In October, zokor caches (1~4 caches in each burrow systems) were excavated, and all food items in each of the burrow systems were collected. Along each burrow system (~10 cm away from the tunnels to prevent potential influences from zokor foraging activities), three 50 cm × 50 cm quadrats were sampled and plants along with the soil in each quadrat were dug up and packed into fiber bags. All plants in the three quadrats were washed, air‐dried, and merged as a single sample (Xie et al. 2014a,b). Each plant from the caches and from the quadrats was identified according to Flora Qinghaiica (Liu et al. 1997), dried at 60°C, and weighed using an analytic balance at a resolution of 0.01 g.

The tastes of most plants were determined according to Chinese Materia Medica Monographs (China Pharmacopoeia Committee 2000). For plant species that were not recorded in the monographs, taste was determined by a taste test. Each plant was independently tasted by five healthy volunteers without a history of smoking or alcohol abuse, and the taste that was noted at the highest frequency was designated the “true” taste. Tastes were classified into one of three groups: bitter, sweet, and other‐tastes (including tasteless, pungent, sour, and other tastes that are difficult to describe).

Ivlev's electivity index (*E*_*i*_) (Ivlev 1961) was used to quantify diet selection. *E*_*i*_ was defined as follows: *E*_*i*_ = (*r*_*i*_ − *p*_*i*_) / (*r*_*i*_ + *p*_*i*_), where *r*_*i*_ and *p*_*i*_ are the relative abundances of a plant species in the caches and quadrats, respectively. According to Ivlev's selection index, *E*_*i*_ ranges from −1 to 1, and *E*_*i*_ **< 0 indicates a negative preference, while *E*_*i*_ **> 0 indicates a positive preference. For each burrow system, plants from caches and quadrats were merged into three groups according to taste (bitter, sweet, and other‐tastes). The *E*_*i*_ value for each zokor was estimated for each of the categories.

### Sequencing and genetic variation of *Tas2r* genes

Individual zokors whose burrow systems were surveyed were live trapped using homemade ground arrows. The ground arrow contains a stent, a hexad‐arrow, an inducing string, and an alarm. The trapping principle works as follows: When a zokor comes to plug up the excavated burrow, the hexad‐arrow suddenly knocks into and blocks the burrow behind its body, and at the same time, the alarm is triggered, and then the field worker can catch the animal in time. The necks of trapped zokors were broken, and muscle samples from each zokor were collected and fixed in 95% alcohol. All animal procedures in this study were conducted with ethical approval from the Ethics Committee, Northwest Institute of Plateau Biology, Chinese Academy of Sciences (No. NWIPB–2014–01). No specific permission was required for the activities in this study. The field studies did not involve endangered or protected species.

In total, 36 mouse and 25 human *Tas2r* genes were retrieved from GenBank (the accession numbers were listed in Table S1) to conduct TBlastN searches (e‐value cutoff = 1e‐10) against the zokor genome (over 100X of sequencing depth, unpublished). For each *Tas2r* gene, the best‐hit scaffold was extracted using the cdbfasta program (Lee et al. 2005) was then aligned with the corresponding mouse gene. The primers for amplification were designed according to the flanking sequence of the zokor scaffold using Primer Premier 5 (Table S2).

Total DNA of each zokor sample was extracted using standard phenol–chloroform extraction methods for animal tissues (Sambrook et al. 2001). PCR amplifications were performed in total reaction volumes of 40 *μ*L, containing 100 *μ*mol/L of each dNTP, 0.25 *μ*mol/L of each primer (synthesized by Sangon Ltd., Shanghai, China), 0.5 *μ*L of template DNA, 5 *μ*L Taq buffer, and 2.5 U Taq DNA polymerase (TransGen Biotech, Beijing, China). The reaction mixtures were denatured at 95°C for 5 min and subjected to 35 cycles of 45 sec at 95°C, 1 min at 50–55°C (depending on the primer pair), 1.5 min at 72°C, and a final extension step of 7 min at 72°C. PCR products were purified and sequenced in both directions on an ABI 373 automated sequencer. Sequences were recorded for both strands with an overlap threshold of at least 30%.

The sequences of each segment were inspected by eye, aligned using ClustalX (Thompson et al. 1997), and viewed refined manually in Chromas v2.31 (Chroma Technology Corporation). The variable site information of DNA sequence was counted directly in the MEGA program (Tamura et al. 2011). The DNA alignments were then translated into AA (amino acid) sequences using the dna2pep program (Python scripts) (Wernersson 2006), and polymorphic sites were counted using MEGA. The DNA and AA alignments were also imported into DAMBE (Xia 2013) to identify individuals with the same genotype (here the diplotype, i.e., matched haplotype pairs, was used to represent the genotype of an individual because the *Tas2r* genes are diploid). The frequency of each genotype was then recorded at both the DNA and AA levels.

### Statistical analyses of phenotype–genotype relationships

Before statistical analyses, the normality of *r*_*i*_, *p*_*i*_, and *E*_i_ values was assessed using the one‐sample Kolmogorov–Smirnov test. Many indices, such as the *E*_i_ of bitter plants (*Z *=* *1.523, *N *=* *56, *P *=* *0.019), deviated significantly from normality. Thus, nonparametric statistical methods were used for the analyses. The plant amounts of each taste were compared between caches and quadrats using the Wilcoxon signed rank (two‐related samples) test. These tests were used to determine whether zokors show a universal (on the whole) food selection behavior in their caches. Chi‐square tests were also performed to determine whether zokor individuals show a random distribution with respect to bitter plant acceptance.

According to population genetics theory, the frequency of a genotype represents its relative fitness under natural selection (Gillespie 1998). In order to guarantee sufficient sample sizes in comparing different genotype groups, for each gene, we only divided the individuals into two groups: major genotype group and minor genotype group. Zokors were divided into two groups according to their genotype at the DNA level: the major group with the predominant diplotype and the minor group with diplotype(s) other than the predominant diplotype. Mann–Whitney tests were implemented to compare the mean bitter plant *E*_*i*_ values between the groups. Additionally, the zokors were divided into two groups according to their genotype at the peptide level: the major group with the predominant diplotype and the minor group with diplotype(s) other than the predominant diplotype.

In addition to a major indel site, there were six single‐nucleotide polymorphisms (SNPs) in *zTas2r134*. In this study, only the indel site was considered owing to its more obvious effects on function. The 56 zokors were arbitrarily divided into two groups: the major group with two intact alleles at the indel site and the minor group with at least one pseudogene. All statistical analyses were executed in SPSS 20.0 (IBM Corp, Armonk, NY, USA).

## Results

### Food composition

We excavated 56 burrow systems and collected all available food items in the temporary overwinter caches and in the vicinity quadrats for each burrow system. We identified 80 species, as well as one group of grasses (Gramineae) in either the caches or the quadrats, of which 33, 32, and 16 plants were perceived as bitter, sweet, and other‐taste, respectively (Table S3). The quadrats were dominated by other‐taste plants (68.06 ± 25.58%; median, 70.36%), and included lower frequencies of bitter (16.91 ± 20.15%; median, 11.41%) and sweet (15.03 ± 15.89%; median, 7.99%) plants. In contrast, bitter (38.30 ± 30.53%; median, 34.33%) and sweet (54.76 ± 32.14%; median, 56.11%) plants dominated the caches, with rather small proportions of other‐taste plants (6.94 ± 9.98%; median, 3.41%) (Table S4).

### Food selection

The Wilcoxon signed rank test showed that percentages of bitter and sweet plants (by weight) in caches were significantly higher than those in quadrats (*N *=* *56, *P *<* *0.001). In contrast, the other‐taste plants were significantly less frequent in caches than in quadrats (*N *=* *56, *P *<* *0.001) (Fig. 1C). The selection index of bitter plants (*E*_bitter_) ranged from −1 to 0.98 (0.31 ± 0.64; Fig. 1D). Of the 56 zokors, 41 showed a preference for bitter plants, and 15 were inclined to avoid bitter plants. The chi‐square test showed that the ratio of individuals that preferred bitter plants to those that avoided bitter plants deviated significantly from 1:1 (*χ* ^*2*^ = 12.071, *P = *0.001, df = 1), that is, the zokor population was dominated by individuals with a preference for bitter food.

### *Tas2r* genes

We identified 26 potential *Tas2r* genes via a blast search with 36 mouse and 25 human *Tas2r* genes on the zokor genome (unpublished). Using primer pairs designed according to the flanking sequences, we successfully sequenced all 26 genes for the 56 zokors. Based on the peptide sequence similarity with mouse *Tas2r*, we named the genes *zTas2r106*,* zTas2r108*,* zTas2r110*,* zTas2r113*,* zTas2r115*,* zTas2r116*,* zTas2r117*,* zTas2r118*,* zTas2r119*,* zTas2r120*,* zTas2r121*,* zTas2r122*,* zTas2r124*,* zTas2r125*,* zTas2r126*,* zTas2r129*,* zTas2r131*,* zTas2r134*,* zTas2r135*,* zTas2r136*,* zTas2r137*,* zTas2r138*,* zTas2r139*,* zTas2r143*,* zTas2r144*, and *zTas2r3‐like*. The original sequence alignments are provided in the Supplementary Materials (Data S1).

### Genetic variation

The lengths of the genes ranged from 879 to 1032 bp, and 23 genes showed one or more polymorphic site(s). At the amino acid (AA) level, 18 loci showed at least one polymorphic site. A total of 94 nucleotide polymorphisms were detected, most of which were synonymous or nonsynonymous SNPs. For *zTas2r139*, all individuals had a premature stop codon (i.e., a nonsense mutation) in both alleles; for *zTas2r138*, one individual had a premature stop codon in both alleles. At sites 380–386 of *zTas2r134*, some GG sites were replaced with TTGAGGA (resulting in a pseudogene) (Table 1, also see Data S1 for details).

**Table 1:** Information of 26 Tas2r genes of zokors (Vn, variable site number; Gn, genotype or diplotype number; Mn, frequency of the major genotype)

Gene	DNA level				AA level			
	Length	Vn	Gn	Mn	Length	Vn	Gn	Mn
*zTas2r106*	927	1	3	36	308	0	1	56
*zTas2r108*	894	1	3	34	297	1	3	34
*zTas2r110*	954	0	1	56	317	0	1	56
*zTas2r113*	930	5	10	24	309	2	6	24
*zTas2r115*	936	3	5	37	311	0	1	56
*zTas2r116*	930	10	10	20	309	6	9	20
*zTas2r117*	939	6	14	11	312	4	11	11
*zTas2r118*	879	0	1	56	292	0	1	56
*zTas2r119*	927	2	4	36	308	1	2	55
*zTas2r120*	957	8	5	17	318	4	5	17
*zTas2r121*	918	6	3	54	305	3	3	54
*zTas2r122*	951	2	3	47	316	0	1	56
*zTas2r124*	912	3	5	31	303	2	4	33
*zTas2r125*	930	6	22	9	309	5	14	11
*zTas2r126*	927	3	9	18	308	1	2	48
*zTas2r129*	936	8	21	14	311	4	11	24
*zTas2r131*	957	3	6	26	318	0	1	56
*zTas2r134* a	891/896	1	2	32	296/134	1	2	32
*zTas2r135*	966	5	7	36	321	4	7	36
*zTas2r136*	912	4	10	25	303	2	4	41
*zTas2r137*	951	5	8	28	316	3	4	52
*zTas2r138*	1032	6	12	17	343/323	5	12	17
*zTas2r139*	951	2	10	47	270	2	3	52
*zTas2r143*	885	0	1	56	294	0	1	56
*zTas2r144*	963	1	3	54	320	0	1	56
*zTas2r3like*	912	3	3	28	303	2	3	28

a Only the indel site in *zTas2r134* was considered and was viewed as one variable site both at DNA and AA levels.

### *Tas2r* genes vs. food selection

**Figure 2:**
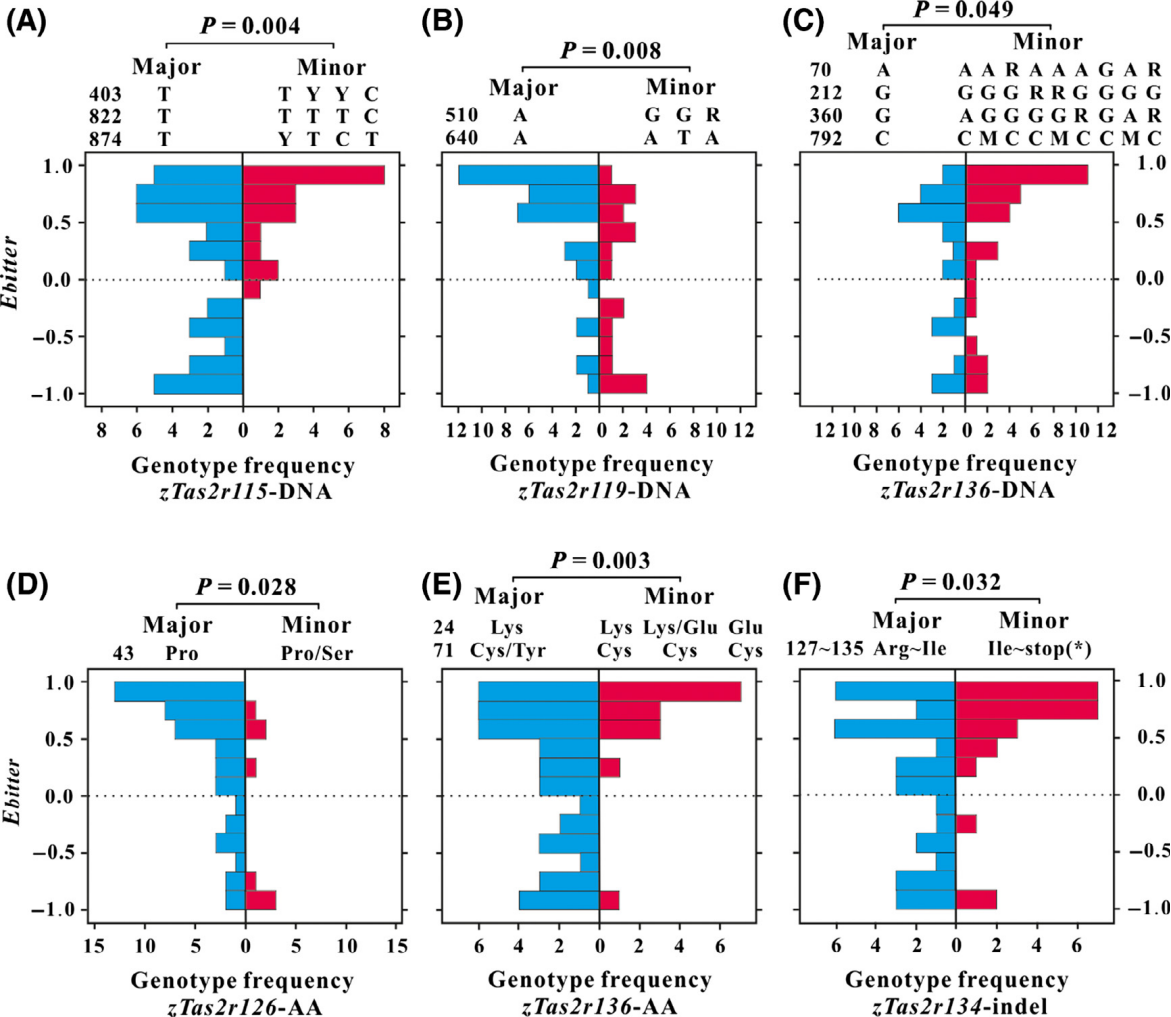
Population pyramid of *E*_bitter_ (selection index of bitter plants) of major and minor genotypes of the genes that have significant relationships with bitter plant selection. (A, B, and C) at DNA sequence level; (D and E) at amino acid (AA) sequence level; (F) only considering the indel site. The numbers before the major DNA/AA genotypes show the mutation sites of DNA/AA sequences; the blue and red bars show the frequency distributions of the 56 zokors with major and minor genotypes, respectively.

Based on Mann–Whitney tests, at the DNA level, *zTas2r115* (*P *=* *0.004), *zTas2r119* (*P *=* *0.008), and *zTas2r136* (*P *=* *0.049) showed significant deviations in *E*_bitter_ values between the major genotype and other genotype(s) (Fig. 2A–C). At the AA sequence level, *zTas2r126* (*P *=* *0.028) and *zTas2r136* (*P *=* *0.003) showed significant deviations in *E*_bitter_ values between the major genotype and other genotype(s) (Table 2; Fig. 2D and E). For *zTas2r134*, the major genotype showed significant (*P *=* *0.032) deviations in *E*_bitter_ values from those of the other genotypes (Fig. 2F). The mean rank values for each group of genes are listed in Table 2, which showed that (without regard to mutation type) the bitter plant acceptance levels were lower for the major genotypes of *zTas2r115*,* zTas2r134*, and *zTas2r136* than for the minor genotypes. In contrast, the major genotypes of *zTas2r119* and *zTas2r126* had higher bitter plant acceptance levels than the minor genotypes. The genotype distributions of each zokor for each gene were listed in Table S5 (DNA level) and Table S6 (AA level).

**Table 2:** Statistical results of zokor *Tas2r* genes that have significant relationships with bitter plant selection (*E*_bitter_, selection index of bitter plant; Major, major group; Minor, minor group)

Mutation type	Gene	Frequency Major: Minor	Mean rank of *E*_bitter_		*Z* value	*P* value
			Major	Minor		
DNA	*zTas2r115*	37 : 19	23.97	37.32	−2.899	0.004
	*zTas2r119*	36 : 20	32.83	20.70	−2.668	0.008
	*zTas2r126*	38 : 18	26.53	32.67	−1.316	0.188
	*zTas2r136*	25 : 31	23.72	32.35	−1.970	0.049
AA	*zTas2r115*	56 : 0	–	–	–	–
	*zTas2r119*	55 : 1	28.35	37.00	−0.526	0.599
	*zTas2r126*	48 : 8	30.43	16.94	−2.166	0.028
	*zTas2r136*	41 : 15	24.60	39.17	−2.961	0.003
Indel	*zTas2r134*	32 : 24	24.45	33.90	−2.144	0.032

## Discussion

### Phenotype–genotype associations for taste preferences

Our results showed that, consistent with the results of our previous study (Xie 2014), zokors do not forage randomly; rather, they generally prefer sweet and bitter plants and avoid other‐taste plants. On average, the weight of bitter and sweet plants were 2.3‐ and 3.6‐fold in caches than in quadrats, while other‐taste plants were much less heavy in caches. It should be noted that there was considerable variation in bitter plant selection among individuals, indicating variation in food selection strategies. We also detected high levels of polymorphism in *Tas2r* genes, which provides a basis for analyzing genotype–phenotype associations.

Variation in 5 *Tas2r* genes was associated with bitter food selection. Importantly, these genes could be classified into three types according to their mutation types. For *zTas2r115* and *zTas2r119*, nearly all of the observed mutations were synonymous (except for one nonsynonymous SNP in *zTas2r119*). Synonymous SNPs are generally not expected to affect protein function because they do not alter the amino acid sequence. However, as they define the mRNA secondary structure and stability and affect the rates of translation, folding, and posttranslational modifications of nascent polypeptides, functional affects have been repeatedly established by recent studies (Kimchi‐Sarfaty et al. 2007; Shabalina et al. 2013). As *Tas2r* genes do not contain introns, we inferred that synonymous SNPs in the exons of these genes may influence function.

At the AA sequence level, *zTas2r126* and *zTas2r136* showed significant relationships with bitter plant selection. Although *zTas2r136* was also related to bitter plant selection at the DNA sequence level, the association was far more significant at the AA sequence level (*P *=* *0.049 vs. *P *=* *0.003). For *zTas2r126*, we did not detect any phenotype–genotype relationships (*Z *=* *‐1.316, *P *=* *0.188) at the DNA level. Hence, we deduce that the genotype–phenotype relationships for the two genes were limited to nonsynonymous SNPs (i.e., they depended on the AA sequence). For *zTas2r136*, the nonsynonymous SNPs were Lys24Glu and Cys71Tyr; for *zTas2r126*, the nonsynonymous SNP was Pro24Ser. Each of these SNPs had a strong effect on the biochemical properties of the loci (Lys24Glu, from alkaline to acid; Cys71Tyr, from sulfur to aromatic; Pro24Ser, from nonpolar to polar). We suggest that these mutations influenced the function of the relevant peptides by affecting their spatial structures.

Mutations in *zTas2r134* could be classified as pseudogenization (indel) type. Pseudogenization is an indicator of whether a gene is functional and is not rare for *Tas2r* (Li and Zhang 2013; Zhao et al. 2015). Previous studies have shown that *zTas2r134* is intact in the mouse (*Mus musculus*), rat (*Rattus norvegicus*), and pig (*Sus scrofa*) (Wu et al. 2005; da Silva et al. 2014). Moreover, the expression of this gene is prominent in gastrointestinal tissues and the tongue (da Silva et al. 2014). These results suggest that *zTas2r134* has functional integrity in many mammals. Pseudogenization at the intraspecific level is rarely reported (Sugawara et al. 2011). Our results provide evidence for a relationship between pseudogenization and ecological phenotypes at the intraspecific level.

### Potential mechanisms underlying the observed genotype–phenotype associations

Animals other than humans are not able to convey their perception of taste for a particular compound; accordingly, we can only speculate about *Tas2r* functions based on their human orthologs or closely related paralogs. Based on *Tas2r* genes of mice and humans in NCBI and phylogenetic analyses (Conte et al. 2003), we identified human orthologs of the five zokor genes as follows: *zTas2r115* is orthologous to *hTas2r14*,* zTas2r119* to *hTas2r1*,* zTas2r126* to *hTas2r41*,* zTas2r134* to *hTas2r16*, and *zTas2r136* to *hTas2r46*. According to a previous study (Meyerhof et al. 2010), human bitter taste receptors have different receptive ranges for bitter compounds: Of the five *Tas2r* genes examined in this study, *zTas2r115* and *zTas2r136* were broad spectrum receptors, that is, they showed extremely wide receptive ranges; *zTas2r119* showed an intermediate receptive range, while *zTas2r134* and *zTas2r126* had very narrow receptive ranges. Interestingly, there appeared to be a substantial correlation between the significance levels for phenotype–genotype relationships and the receptive ranges for the five genes. For example, *zTas2r115* and *zTas2r136* had the widest receptive ranges and were most strongly associated with bitter plant selection (for *zTas2r136*, only correlations at the AA level were considered). As the plants contain complex bitter compounds, we suggest that the two genes that can sense various bitter substrates played a pivotal role in shaping the observed phenotype–genotype associations (Fig. 2A and E).

There was incongruence in the bitter plant preference between the major and minor genotypes for the five genes. The major *zTas2r115*,* zTas2r134*, and *zTas2r136* alleles had lower bitter plant acceptance levels than the minor genes. Although we cannot conclusively determine whether synonymous (*zTas2r115*) or nonsynonymous (*zTas2r136*) mutations were related to less sensitive phenotypes than the major genotypes, it is clear that pseudogenized genotypes (*zTas2r134*) are associated with a weaker sensitivity to bitter taste than the intact major genotypes. As most mutant genotypes for all the three genes were associated with positive *E*_bitter_ values (Fig. 2A, E, F), we inferred that zokors with mutation in these loci had higher tolerance levels to bitter plants owing to their weak bitter sensitivity.

Interestingly, however, the zokors with major *zTas2r119* and *zTas2r126* genotypes preferred bitter plants to a greater extent than zokors with mutations in these genes. There are two potential explanations for this phenomenon. First, the sensitivity to bitter foods was weaker for the major genotypes than the mutant genotypes. Second, the major genotypes promote active, rather than passive (not able to refuse bitter foods because of weaker bitter sensitivity), foraging of bitter foods, which may improve survival (because of wider food resource ranges). Although bitter plants may be poisonous, some may also be nutritious; as a result, zokors might take some bitter compounds as chemical clues for collecting nutrient plants. Our previous study showed that zokors prefer poisonous plants, such as *Stellera chamaejasme*, and they are apparently well suited to ingesting toxic chemicals or digestion‐reducing chemicals (Lin et al. 2012; Xie 2014). We suggest that the strong ability to consume with poisonous plants confers an advantage with respect to actively foraging bitter plants via the two genes.

Foraging ecology is highly complex for herbivores. In addition to the taste of chemical compounds, many plant traits, such as abundance and physical defenses, have the potential to influence foraging behavior (Pyke 1984; Carmona et al. 2011). In this study, owing to difficulties related to field work, we classified plants roughly according to their tastes, and did not consider variation in other physical, chemical, or ecological characteristics among plants. Additional laboratory studies, for example, cafeteria tests, are necessary to further elucidate the relationship between diet selection and variation in *Tas2r* genes in plateau zokor. In the next step, we will firstly test whether the effect of a given SNP or pseudogenization event that is associated with an increased preference for bitter plants could be explained by a decrease in the response of the receptor to those bitter ligands.

In conclusion, our study demonstrated variation in bitter plant selection among zokor individuals. Variation was also observed at the DNA and AA sequence levels in genes related to taste perception. There were strong relationships between bitter plant selection and genotype for five *Tas2r* genes. Bitter plant selection was related to synonymous SNPs in *zTas2r115* and *zTas2r119*, nonsynonymous SNPs in *zTas2r126* and *zTas2r136*, and pseudogenization in *zTas2r134*. The minor genotypes of *zTas2r115*,* zTas2r134*, and *zTas2r136*, and the major genotypes of *zTas2r119* and *zTas2r126* were associated with a preference for bitter plants, indicating that zokors actively and passively accept bitter foods.

## Data Accessibility

The original sequence alignments of 56 zokors, respectively, for the 26 *Tas2r* genes were uploaded as online (Data S1).

## Conflict of Interest

None declared.

## Supporting information


**Table S1.** GenBank accession numbers for human and mouse *Tas2r* sequences that were used for blast researching the zokor *Tas2r* genes.
**Table S2.** Primers (5′ to 3′) for amplifying and sequencing the *Tas2r* genes of zokors.
**Table S3.** Plants found in zokor over‐winter caches or vicinity quadrats and their tastes as well as overall appearance frequencies in the 56 burrow systems.
**Table S4.** Composition (weight percentage) of plants with different tastes in caches and quadrats.
**Table S5.** Food selection and genotype distribution at the DNA level of the 56 zokors. The major genotype and minor genotype(s) were marked as 1 and 0, respectively.
**Table S6.** Food selection and genotype distribution at the AA level of the 56 zokors. The major genotype and minor genotype(s) were marked as 1 and 0, respectively.

**Data S1**. Original sequence alignments of 56 zokors respectively for the 26 *Tas2r* genes.
